# Have medical students’ attitudes towards clinical communication skills changed over a 12- year period? A comparative long-term study

**DOI:** 10.1186/s12909-019-1915-1

**Published:** 2020-01-10

**Authors:** Tore Gude, Reidar Tyssen, Tor Anvik, Hilde Grimstad, Are Holen, Anders Baerheim, Per Vaglum, Lise Løvseth

**Affiliations:** 1 Department of Behavioural Sciences in Medicine, Institute of Basic Medical Sciences, Faculty of Medicine, University of Oslo, POB 1111 – Blindern, N-0317 Oslo, Norway; 2 Department of Community Medicine, Faculty of Health Sciences, University of Tromsø, Tromsø, Norway; 3 Department of Public Health and General Practice, Faculty of Medicine and Health Sciences, Norwegian University of Science and Technology, Trondheim, Norway; 4 Department of Mental Health, Faculty of Medicine and Health Sciences, Norwegian University of Science and Technology, Trondheim, Norway; 5 Department of Global Public Health and Primary Care, Faculty of Medicine and Dentistry, University of Bergen, Bergen, Norway; 6 Dept. Research and Development, Division of Psychiatry, St Olavs’ University hospital, Box 3250 Torgarden, 7006 Trondheim, Norway

**Keywords:** Medical students, Communication skills, Attitudes, Doctor-patient relationship

## Abstract

**Background:**

Attitudes towards learning clinical communication skills at the end of medical school are likely to reflect the students’ training and motivation for the continued development of their skills as doctors. Students from two Norwegian medical schools, one with a traditional, and the other with an integrated curriculum, were approached in 2003 and 2015; with regard to changes in students’ attitudes towards acquiring communication skills in two diverse learning environments. This comparison might reveal the effects of the training programs from a long-term perspective, as neither of the medical schools made any major curriculum changes within the study period.

**Methods:**

The samples comprised final-year medical students. Two separate cross-sectional surveys performed 12 years apart (2003 and 2015) used items from the Communication Skills Attitude Scale in addition to age and gender. The traditional curriculum included only theoretical teaching and no contact with patients was made during the first 2 to 2.5 years of medical school. However, the integrated curriculum combined training in theoretical and clinical communication skills with early patient contact from the beginning.

**Results:**

Attitudes improved from the first to the second survey at both schools, however, students from the integrated school reported more positive attitudes than those from the traditional school. Female students from the integrated school contributed the most to the difference in attitudes in both surveys.

**Conclusions:**

Students in both traditional and integrated curricula improved their attitudes from the first to the second assessment. However, compared with the traditional curriculum, the integrated one fostered even higher levels of positive attitudes towards acquiring communication skills, and a pronounced influence was observed on female students. These findings suggest that an educational program with greater emphasis on improving attitudes among male students may be required.

## Background

Clinical communication skills are crucial for making correct diagnostic assessments [1] providing adequate treatment [2], obtaining patient compliance [3], and improving patient satisfaction [4] . Curriculum developers have been concerned about medical students’ attitudes towards learning communication skills for many years [5]. In particular, the number of patients complaining to medical authorities about their doctor, often reflecting the doctors’ unacceptable communication styles and poor behavior [6] has been increasing. Accordingly, medical schools should attempt to foster more positive student attitudes towards acquiring these skills and enhance their students’ professionalism [7] in ways that will translate into their future work as doctors for the benefit of both patients [8] and society.

Since the late 1990s, according to Silverman, clinical communication skills education have come of age and the increasing acceptance of this type of education as a formal component of the medical curriculum has been documented, however, several challenges remain [9].

The content, structure, number of hours and timing of communication training in the curriculum are assumed to be relevant to the outcome and to have a bearing upon the students’ motivation for further acquisition of clinical communication skills. In addition to the ‘official’ curriculum design, the hidden curricula students are exposed to during their years in medical school may affect their attitudes, indeed also attitudes towards clinical communication, both negatively and positively [9]. Attitudes towards the importance of clinical communication as part of the doctor-patient relationship in the society and in the mass media over the 12-year study period, should also be considered an impact factor in regard to attitudes among medical students.

The preclinical part of the traditional medical curriculum includes only theoretical teaching; no contact with patients is made during the initial 2 to 2.5 years of learning. This used to be the common educational model for medical curricula in many countries, including Norway. In recent decades, however, major efforts have been made worldwide to develop integrated models in which both theoretical and clinical communication training are included as a common ingredient in all medical teaching from the beginning, continuing throughout the entire curriculum.

In their book: Doctors Talking to Patients, Byrne and Long account for their interest in doctor-patient interaction by making a large number of recordings from consultations performed as early as the 1970s [10]). Although they lacked the proper analytical tools, their work shed light on this very important topic. According to Pereira Grey, Byrne and Long were among the first to address the issue of communication between doctors and patients as a necessary and sufficient condition for how consultations should be carried out [11].

Regardless, concerns remain about the optimal method for teaching communication skills in medical schools [9]. Comparing student attitudes using cross-sectional surveys over a long time span could indicate stability or changes in regard to learning communication skills. In addition, understanding the influence from different types of curricula (in the present study: traditional vs integrated) could provide an estimate of the likely influence of differences between the two types of teaching environments.

Positive attitudes are well known to be necessary to obtain skills for performing specific tasks. With a lack of continuous training throughout the curriculum, skills and presumably attitudes often deteriorate. Therefore, the focus of the present study is on attitudes students carry with them upon leaving medical school. A comparison of attitudes towards learning clinical communication skills at this point would optimally reflect the training outcome and indicate student motivation for the continued development of their skills as doctors.

In 2003, we surveyed two Norwegian medical schools with traditional and integrated curricula, respectively. The attitudes of the medical students in the school with the integrated curriculum tended to be more favorable throughout all school years and significant differences between schools were observed in the final year [12]. The results also demonstrated that female students generally reported more positive attitudes than male students. Based on these results, the difference in attitudes between the students in the traditional and integrated schools could be attributed to how the two curricula were organized.

Concerning the gender aspect, we found in an earlier study within our research group that female students (young doctors) improved their observed communication skills significantly, while male peers did not show this improvement from end of medical school to the end of an obligatory internship 2 years later [13]. However, whether this finding can be linked to more positive attitudes among females than among males, remains unclear, as the data collected for the present study does not address this issue. Another possible factor that can affect student attitudes is well-being within the school context; however, in a recent study, no such relationship was found [14].

The findings from 2003 led to three pertinent questions. First, are attitudes towards learning clinical communication skills stable over many years? Second, is the difference in attitudes between the 2003 curricula a random finding? Third, were gender issues in the training within medical school overlooked?

A replicated study could test whether attitudes are stable over many years in a persistent learning environment. Further, if the 2003 findings about differences between curricula could be confirmed at a later time point, reasons would exist to point to the integrated curriculum being better suited than a traditional curriculum in developing positive attitudes towards communication skills training.

In 2015, we re-administered the 2003-survey on final-year students in the same two medical schools. The basic structure of the two curricula remained the same over these 12 years, with some minor faculty changes only; therefore, we presumed that the differences between the two schools would be the same as those 12 years earlier. Given the increased awareness of the importance of clinical communication in the medical society and in the general population, one would expect that student attitudes towards clinical communication have become increasingly positive over this period.

Against this background, we proposed the following research questions:

1. Did positive attitudes towards clinical communication, independent of curriculum, increase from 2003 to 2015?
2. Has the impact on attitudes from the curriculum model demonstrated in 2003 been stable or changed over the 12-year period?
3. What are the effects of age and gender on differences in student attitudes?

## Methods

### Design

This study used a long-term comparative survey design. In 2003, students in two of the four Norwegian medical schools were approached. Twelve years later, a new student cohort in the same two medical schools was investigated using the same tools for testing the student attitudes towards learning clinical communication skills.

### Sample

In 2003, 94 (70%) of 135 and 46 (50%) of 91 final-year traditional and integrated medical school students, respectively, responded to the survey. The gender distribution was 61% women with a mean age of 24.5 + 3.2 years in the traditional school and 63% women with a mean age of 24.0 + 2.8 in the integrated school.

In 2015, 81 (56%) of 144 and 88 (78%) of 113 final-year traditional and integrated school students, respectively responded. The gender distribution was 72% women, with a mean age of 24.7 + 3.1 years in the traditional school; and 70% women with a mean of age 25.1 + 3.1 years in the integrated school. The change in gender proportions from the first to the second cross-sectional studies was not statistically significant (not overlapping 95% confidence intervals).

### Curricula descriptions

The basic structure of the two curricula remained the same over the 12 years with only some minor faculty changes. Both medical schools had 6-year curricula, however, their contents, length and timing in regard to the communication training differed (Table 1).

**Table 1:** Characteristics of curriculum schedules in communication skills training at two medical schools

Type of curriculum		Integrated	Traditional
Number of hours for training in clinical communication skills	Start-phase (first 2 years)	24	0
	Mid-phase (3rd–5th years)	16	48
	Last year	5	16
	Total	45	64
Practical work and outplacement in hospitals or at GPs	Start-phase	90 (GP)	0
	Mid-phase	135 (GP 550 (Hospital)	6 (GP) 480 (Hospital)
	Final year	0	140 (GP)
	Total	775	626

GP general practitioner

The initial theoretical preclinical phase in the traditional curriculum was curtailed from 2.5 years in 2003 to 2 years in 2015. This part of the curriculum did not show any systematic changes concerning communication training and patient contact, but the 3.5-year duration of the clinical curriculum in 2003 was increased to 4 years in 2015. During this last part of the curriculum, a cognitive approach model was used during training to teach how patients should be addressed. Training for how ‘to break bad news’ to patients was also given. In addition, the students received specific communication training in hospital (internal medicine) and general practice settings.

In both 2003 and 2015, the integrated curriculum used a problem-based learning model involving early patient contact from the very first days to encompass both preclinical and clinical subjects. Within the first 2 years of this curriculum, students undergo an intensive, closely supervised communication skills training course. The mandatory training includes role- play among the students, and ends with a clinical communication exam using simulated patients. The students also receive somatic clinical training alongside the communication training in the integrated curriculum. As in the traditional school, students in the integrated curriculum also received practical training in both general practice and hospital settings. Table 1 shows the number of hours allocated to the theoretical and practical training in clinical communication skills at the two schools. The admission criteria for the two medical schools remained unchanged during the 12-year period.

### Instrument - dependent variable

Data gathered from the Communication Skills Attitude Scale (CSAS) [15] at the two time points (12 years apart) were compared. The questionnaire was originally composed of 26 items: 13 positive and 13 negative. However, for the purposes of the present study, four items from both surveys that correlated the highest with the total score in 2003 were used: items 1, 7, 21 and reversed item 24. The limited number of items used was due to logistic constraints as part of a larger study.

In two of the four items, minor alterations in the wording were made, which presumably did not influence the assessment of attitudes. In 2003, ‘In order to be a good doctor, I must **have** good communication skills’ was changed to ‘In order to be a good doctor, I must **master** good communication skills’ in 2015. In 2003, ‘Learning communication skills **is really** useful’ was changed to ‘Learning communication skills **is** useful’ in 2015. The other two: ‘Learning communication skills is interesting’, and ‘I find it difficult to take learning communication skills seriously had the same wording on both occasions. The four items (Cronbach’s alpha = .64) were considered representative for the whole instrument as used in the dataset from 2003 (Cronbach’s alpha = .85). In the 2015 dataset, Cronbach’s alpha was .63 for the four items. All items were coded on a five-point Lickert scale from ‘1 = do not agree’ to ‘5 = agree’, where the last item was reversed before calculating the mean score.

To test the psychometric properties and robustness of this abbreviated scale, we performed a principal component analysis (PCA). In both datasets the Kaiser-Meyer-Olkin fit-index (KMO) was .67, and a one-factor solution emerged in the scree plot (eigenvalue > 1, covering slightly above 50% of the variance). The factor-loadings in the 2003 and 2015 datasets for all four items were in the range of .83 to .37, and.84 down to .58, respectively. Although the KMO-index could have been higher, these results indicate that the four-item scale demonstrated acceptable robustness even with the slightly different wording between the two surveys.

Gender was coded as female = 1; and male = 2, while age was recorded as a continuous variable.

### Statistics

Means, correlations, one-way analysis of variance (ANOVA), reliability analysis and PCA were performed using SPSS software (v.22.0: IBM SPSS, Armonk, NY, USA). Cohen’s d was calculated by dividing the differences in scores with the average of the standard deviations.

## Results

A significant increase in positive attitudes was detected in both schools from 2003 to 2015, as the 95% confidence intervals of the means CSAS scores did not overlap. This change was most prominent in the integrated school (t = 2.59 [df = 132], *p* = .01), but a significant improvement was also observed in the traditional school a (t = 2.15 [df = 173], *p* = .04). The mean CSAS scores from the integrated and traditional schools showed a significant difference in both 2003 (4.39 + .52 vs. 4.13 + .63, F = 5.60, *p* < .05), and 2015 (4.62 + .47 vs. 4.33 + .66, F = 12.75, *p* < .001) clearly favouring the integrated school (moderate effect sizes d = .46 and d = .53, respectively: see Table 2). Female students in the integrated school had significantly higher levels of CSAS scores in both assessments than those in the traditional school. This was demonstrated by upper-moderate effect sizes (i.e., d = .66 in 2003 and .63 in 2015). These differences between schools did not emerge among the male students (Table 2).

**Table 2:** Mean scores/differences on CSAS between two medical schools in last year of the curriculum

Survey year	2003		2015		Difference between schools	
School	Integrated school (*N* = 46)	Traditional school (*N* = 94)	Integrated school (*N* = 88)	Traditional school (*N* = 81)	2003	2015
CSAS - mean	4.39 (.52)	4.13 (.63)	4.62 (.47)^#^	4.33 (.66)^§^	F = 5.60* d = .46	F = 12.75*** d = .53
Gender-wise CSAS	♀ 4.47 (.36) (*N* = 24) ♂ 4.30 (.65) (*N* = 22) F = 1.28 n.s.	♀ 4.16 (.58) (*N* = 54) ♂ 4.09 (.71) (*N* = 40) F = .32 n.s.	♀ 4.69 (.41) (*N* = 67)^#^ ♂ 4.42 (.60) (*N* = 21) F = 5.50* d = .54	♀ 4.39 (.54) (*N* = 56)^§^ ♂ 4.19 (.71) (*N* = 25) F = 1.20 n.s.	F = 5.78* d = .66 F = 1.29 n.s.	F = 12.20** d = .63 F = 1.34 n.s.

* = *p* < .05, ** = *p* < .01, *** = *p* < .001. ^#^ < .05 within the Integrated school from 2003 to 2015. ^§^ < .05 within the Traditional school from 2003 to 2015. d = Cohen’s d

The bottom F-values indicate gender differences within schools

In 2015, but not in 2003, the between-gender differences within each school significantly favoured female students at the integrated school. No significant difference was observed when controlling for age in the analyses.

## Discussion

Our main finding was that the 2015 cohort of medical students in both schools demonstrated a higher level of positive attitudes towards learning clinical communication than did the 2003 cohort of students. Even though the change was greater in the integrated compared with the traditional school, both displayed an increasing trend of favourable attitudes towards acquiring clinical communication skills over the 12-year period between the two surveys. This result probably reflects general changes within the medical community, and society as a whole, in that doctors are increasingly expected to communicate well and empathetically with their patients.

Another main finding was that the students in the integrated curriculum reported a higher level of positive attitudes than did the students in the traditional curriculum at both assessments. Thus, the differences found in 2003 should not be viewed as an arbitrary finding, but rather, to be related to differences in the characteristics between the two curricula.

The higher level of positive attitudes in the integrated school was mostly explained by the responses from the female students, which indicate that they may benefit more from the integrated curriculum. This finding highlights some pertinent questions about communication training including ‘Is there a gender-related challenge even for integrated medical curricula’ and ‘Are there gender-related aspects of communication training that have not been adequately addressed?

Prior studies have addressed gender in relation to attitudes towards learning communication skills. Kaufmann et al. showed that female students had more positive attitudes than did males [16]. This finding is consistent with Batenburg, who found that female students scored the same as or higher than male students in regard to professional attitudes before and after a communication skills course [17]. In his review of the literature, Aspegren cited four studies, all of which showed that male students were slower than females in learning communication skills [18]. A possible reason for this could be that the acquisition of these skills includes emotional exposure and requires reflection on the doctor-patient relationship. These traits are often viewed to be more prominent among female students, an assumption being consonant with recent studies [19, 20]. Interestingly, the decline in well-being among female students found in the same dataset (2015) as that used in the present study does not seem to have influenced their attitudes toward communication training negatively, but rather, the contrary [14]. Further efforts appear to be required to enable the male students to achieve similar levels. Our findings did not corroborate that age made any difference; most medical students tend to be in the younger adult bracket. Therefore, their age distribution is limited.

As mentioned above, our interpretations are supported by the results from a study conducted by our research group some years earlier; that study demonstrated that young female doctors improved their communication skills significantly more than did young male doctors from the end of medical school until the end of their postgraduate internship 1.5 years later [13]. These findings are in line with those from other studies that show men to be slower learners than women in this field [18]. Accordingly, these results underscore the fact that medical schools may face a major challenge in improving attitudes towards better acquisition of communication skills among male students. Some of the referenced literature deals with data from doctors, whereas the data in the present study cover final-year medical students; therefore, any possible sample differences are likely to present no or only a minor bias.

The gender distribution in our two populations differed to some degree between the two surveys. The 2015 sample had a higher proportion of women than did the 2003 sample. Even though this difference was not statistically significant, a type I error may have occurred as the female students contributed more towards the between-school differences. However, we would have expected a larger difference in 2015 compared with 2003 if any type I error had been present. The close-to-equal effect sizes for the mean CSAS scores at both assessments, even with somewhat larger F-values (one-way ANOVA), seems likely to represent a lower risk of such errors (Table 2).

The integrated school not only enabled the students to have contact with patients right from the very beginning of training, but also simultaneously provided an intensive training program in communication skills during the initial part of the curriculum. Among students, this may have already fostered a better and earlier understanding of various communication challenges in the first part of their curriculum. Accordingly, these students, especially females, may have developed a greater eagerness to continue learning about communication skills in their post-graduate career.

This study has both strengths and limitations. One strength is that we applied items from the same instrument at both assessments with many years in between. Whether a response rate around 60% can be considered satisfactory depends upon the representativeness of the participating students. Data from an earlier study (the 2003 data used in this study), did not show a difference in gender distribution or in age between responders and non-responders. This reduces the possible risk of a sampling bias.

Another potentially limiting factor is that we based our study on four items derived from a single questionnaire. However, the CSAS instrument has been widely used and has demonstrated satisfactory psychometric properties [19]; this was also found in our datasets. For this reason, our statistical procedures should limit the chance of bias due to item selection. A Cronbach’s alpha of .63 is at the lower limit of being acceptable, but we obtained significant differences even at that value which indicates that the internal consistency of our outcome measure is sufficient. In addition, both PCAs yielded a satisfactory robust one-factor solution with adequate factor loadings.

Can we rely on data derived from a self-report questionnaire? This issue has been addressed in an earlier study [21]. The conclusion was that self-report data is likely to be as reliable as interview data, but when it comes to self-reporting of ones’ own accomplishments, reliability is lacking [22].

Our design did not include continuous data at the individual level throughout medical school and beyond. Instead, we used two cross-sectional datasets from 2003 and 2015 for logistical reasons in a long-term comparative survey design. The absence of longitudinal, individual data would be a problem only if the students in the two samples were very different in terms of their general attitudes towards learning. In this respect, we assumed that, even with a general increased awareness upon communication as such, the students’ personalities, contexts, and life situations did not differ too much between our two surveys. No major selection bias was likely to have influenced one cohort, but not the other in regard to these issues.

## Conclusions

Students in both traditional and integrated curricula improved their attitudes from the first to the second assessment. However, compared with the traditional curriculum, the integrated one fostered even higher levels of positive attitudes towards acquiring communication skills, and a pronounced influence was observed on female students. These findings suggest that an educational program with greater emphasis on improving attitudes among male students may be required.

## Data Availability

The datasets used and/or analyzed during the current study can be available by the corresponding author on reasonable request.

## Abbreviations

ANOVA: (One-way) Analysis of variance
CSAS: Communication Skills Attitude Scale
GP: General practitioner
KMO: Kaiser-Meyer-Olkin
PCA: Principle Component Analysis
SPSS: Statistical Package for the Social Sciences
